# Survivorship of the fixed-bearing medial unicompartmental knee arthroplasty: mean 14-year follow-up in a single medical center

**DOI:** 10.1186/s12891-024-07378-1

**Published:** 2024-04-12

**Authors:** Kung-Tseng Hung, Chun‑Chieh Chen, Yu-Chih Lin, Sheng-Hsun Lee, Chih-Chien Hu, Yu-Han Chang, Pang-Hsin Hsieh, Hsin-Nung Shih, Chih-Hsiang Chang

**Affiliations:** 1 Department of Orthopaedic Surgery, Linkou Chang Gung Memorial Hospital, No. 5, Fuxing St., Guishan Dist, Taoyuan, Taiwan; 2 Bone and Joint Research Center, Chang Gung Memorial Hospital, Taoyuan, Taiwan; 3 Graduate Institute of Clinical Medical Sciences, College of Medicine, Chang Gung University, Taoyuan, Taiwan; 4 College of Medicine, Chang Gung University, Taoyuan, Taiwan

**Keywords:** Unicompartmental knee arthroplasty, Fixed-bearing, Survival, Risk factors

## Abstract

**Background:**

This study aimed to report the long-term survival of fixed-bearing medial unicompartmental knee arthroplasty (UKA) with a mean of 14-year follow-up, and to determine possible risk factors of failure.

**Methods:**

We retrospectively evaluated 337 fixed-bearing medial UKAs implanted between 2003 and 2014. Demographic and radiographic parameters were measured, including pre-operative and post-operative anatomical femorotibial angle (aFTA), posterior tibial slope (PTS), and anatomical medial proximal tibial angle (aMPTA). Multivariate logistic regression analysis was applied to figure out risk factors.

**Results:**

The mean follow-up time was 14.0 years. There were 32 failures categorized into implant loosening (*n* = 11), osteoarthritis progression (*n* = 7), insert wear (*n* = 7), infection (*n* = 4), and periprosthetic fracture (*n* = 3). Cumulative survival was 91.6% at 10 years and 90.0% at 15 years. No statistically significant parameters were found between the overall survival and failure groups. Age and hypertension were significant factors of implant loosening with odds ratio (OR) 0.909 (*p* = 0.02) and 0.179 (*p* = 0.04) respectively. In the insert wear group, post-operative aFTA and correction of PTS showed significance with OR 0.363 (*p* = 0.02) and 0.415 (*p* = 0.03) respectively. Post-operative aMPTA was a significant factor of periprosthetic fracture with OR 0.680 (*p* < 0.05).

**Conclusions:**

The fixed-bearing medial UKA provides successful long-term survivorship. Tibial component loosening is the major cause of failure. Older age and hypertension were factors with decreased risk of implant loosening.

**Supplementary Information:**

The online version contains supplementary material available at 10.1186/s12891-024-07378-1.

## Introduction

Osteoarthritis (OA) and osteonecrosis (ON) of the knee are two common indications for knee arthroplasty procedures [1]. Isolated unicompartmental damage of the knee joint is often an early manifestation in patients with such clinical entities. Operative options include corrective osteotomy, unicompartmental knee arthroplasty (UKA), and total knee arthroplasty (TKA). Operative choice is based on the level of joint damage, alignment, and shared decision making between patients and surgeons.

The advantages of UKA over TKA include the preservation of bone, less invasiveness, faster recovery, and better range of motion [2, 3]. Moreover, the improvement in implant designs, surgical techniques, and biomaterials rendered UKA a reliable procedure for patients [4, 5]. In addition, a British study showed that 47.6% of the patients undergoing knee replacement were candidates for UKA based on radiographic assessment [6]. Despite the reported benefits, UKA has inconsistent results [7–14]. The cumulative survival rate for patients with OA undergoing UKA was only 82% at 10 years [14] compared to 96% at 12 years for patients with ON [11]. However, compared to TKA, UKA had a ten-year survivorship of only 80% with a higher revision rate than TKA of 92 ∼ 93% survivorship in the Norwegian and Finnish registry database [8, 10]. Another Italian registry data also revealed lower survivorship of UKA compared with TKA at 15 years with 81.8% and 93.8% respectively [7].This study aimed to report the survivorship of fixed-bearing medial UKAs at a mean of 14 years follow-up, to identify the causes of implant failure, and to determine the possible risk factors of the failure modes.

## Materials and methods

**Fig. 1:**
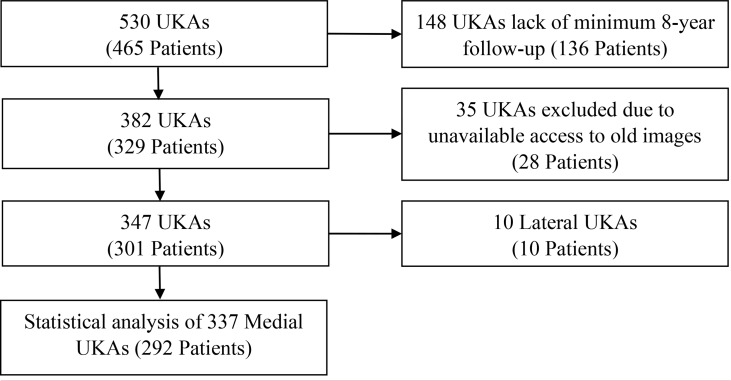
Flow chart of case enrollment

This study was approved by the local Biomedical Institutional Review Board with IRB Approval No.103-3539B. A total of 530 fixed-bearing metal-backed UKA cases (ZUK, previously Zimmer® Unicompartmental High Flex Knee, Zimmer Biomet, Warsaw, IN, USA, now owned by Lima Corporate® or Smith and Nephew®) were performed on 465 patients in our department between January 2003 and August 2014. To evaluate the long-term outcomes, a minimum of 8-year follow-up for patients undergoing UKAs was set. By September 2022, 329 patients corresponding to a total of 382 UKA cases had undergone follow-up in the outpatient department, while a total of 136 patients corresponding to 148 cases did not meet the minimum 8-year follow-up and were thus excluded from the study. Furthermore, after retrieving data for further analysis, 35 UKAs in 28 patients were excluded due to unavailable access to the old images, rendering 347 UKAs with complete data, out of which only ten cases were lateral UKAs. Considering the differences in alignment and surgical techniques compared to medial UKAs, these ten cases were excluded from further analysis. Figure 1 demonstrates the flow chart of case enrollment.

### Patient and surgical characteristics

The indications and contraindications of UKA for patients were listed in Table 1. All surgeries were performed under general anesthesia using standard methods with spacer block technique. In every case, both femoral and tibial components were cemented. After the surgery, all patients were subjected to regular outpatient follow-up at two weeks, four weeks, three months, six months, and then yearly. For implant related issues, implant size of femur, tibia, and insert were recorded. In addition, body mass index (BMI) and major comorbidities such as hypertension and diabetes mellitus were documented.

**Table 1:** Indications and contraindications of UKA

Indications	Contraindications
Isolated compartmental OA or ON	Inflammatory arthritis
Intact cruciate ligaments	Previous surgery of contralateral meniscus
Intact collateral ligaments	
> 90° Range of motion	
< 15° Flexion contracture	
< 15° Passively correctable varus/valgus deformity	
Asymptomatic patellofemoral joint	

### Radiographic evaluation

**Fig. 2:**
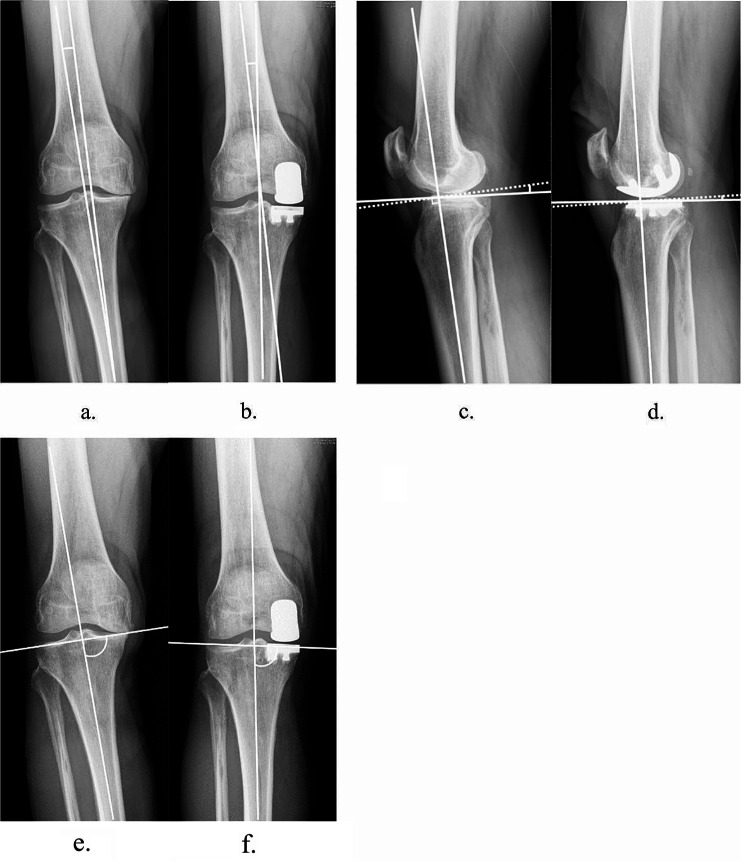
Measurement of pre-operative and post-operative alignments. (**a**, **b**) aFTA (**c**, **d**) PTS (**e**, **f**) aMPTA

Pre-operative and post-operative radiographs including the knee anteroposterior view, lateral view, and Merchant view were collected. Weight-bearing radiographs were obtained within four weeks postoperatively. For patients who presented painless knee, clinical visit without radiograph were made after 1 year post-operatively. Regarding the mechanical factors that might influence surgical outcome, the following parameters were measured: the pre-operative / post-operative anatomical femorotibial angle (aFTA), the posterior tibial slope (PTS), and the anatomical medial proximal tibial angle (aMPTA) (Fig. 2 ). The aFTA was measured by the angle between the anatomical axes of the femur and the tibia. Valgus was recorded as positive value while varus was recorded as negative value. The PTS was defined as the angle between the line perpendicular to the anatomical axes of the tibia and the line connecting the anterior and posterior tibial plateau. The aMPTA was defined as the medial angle between the tibial joint line and the anatomical axis of the tibia. Specifically, the tibial joint line in the post-operative aMPTA was measured as a line parallel to the tibial tray. The correction angles (Δ) were also calculated by subtracting the pre-operative values from the post-operative values, which were then presented in absolute values.

### Statistical analysis

Failure of the procedure was considered at the time when revision surgery for any cause was made or if a periprosthetic fracture developed. The data were expressed as means ± standard deviations (SD). Shapiro-Wilk test was performed preliminarily to assess for normality of data. A life-table showing overall survivorship was constructed. Survival analysis for the OA group and the ON group was demonstrated using the Kaplan-Meier method along with the log-rank test. Independent t-test and Mann-Whitney U test were applied for analysis accordingly. Chi-Square test and Fisher’s exact test were applied for nominal data correspondingly. To figure out possible risk factors of failure, univariate and stepwise multivariate logistic regression analysis were used. Purposeful selection of variables consisting of three steps of iterative process of deleting, refitting, and verifying variables was applied for the stepwise multivariate analysis to improve the chances of retaining meaningful covariates and confounders as recommended by Bursac et al. [15]. The threshold for statistical significance was set at α = 0.05. Data was analyzed using the commercially available statistical software SPSS 20 (IBM Corp., Armonk, NY, USA).

## Results

**Fig. 3:**
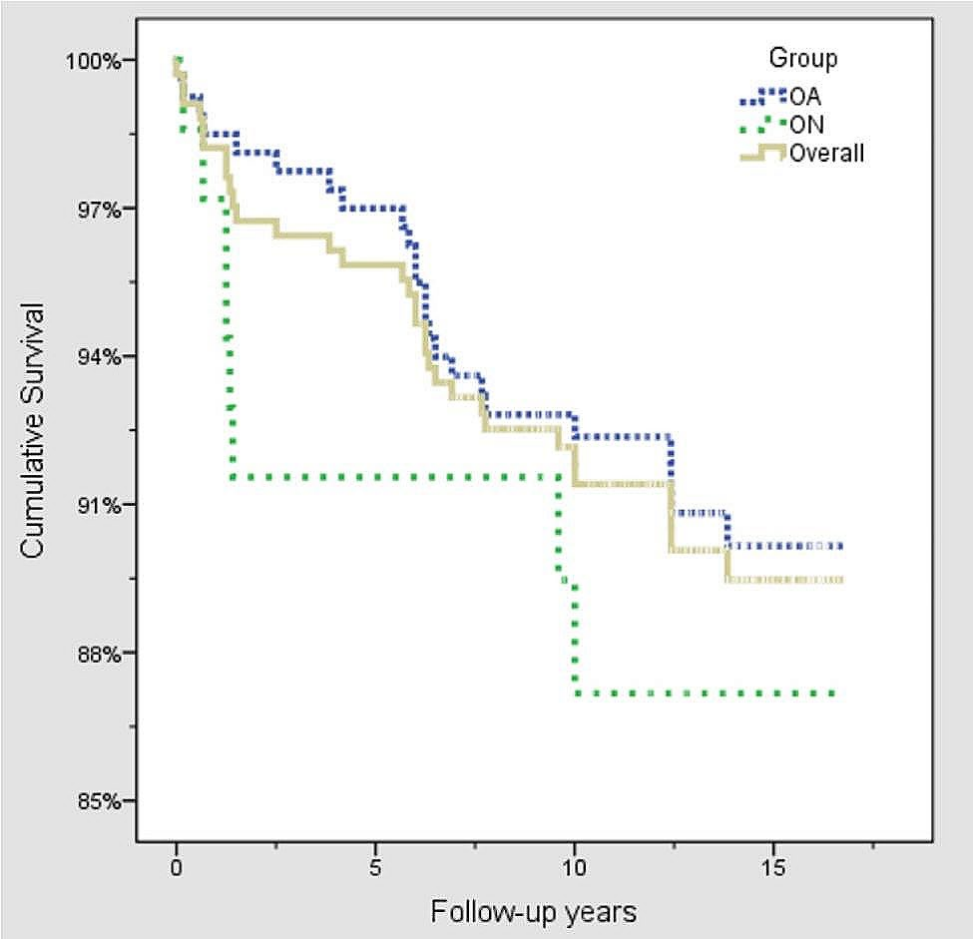
Kaplan-Meier survival analysis between the OA and the ON groups. In the OA group, the cumulative survival was 92.5% at ten years and 90.6% at 15 years, whereas the ON group had a cumulative survival of 88.1% at ten years and 15 years (log-rank test *p* = 0.443)

Table 2 demonstrates the details of the 337 medial UKAs, and no statistically significant differences was noticed. The mean follow-up period was 14.0 ± 4.1 years. Table 3 shows a life-table for the remaining 337 medial UKAs. The cumulative survival was found to be 91.6% at ten years, and 90.0% at 15 years. Figure 3 shows a Kaplan-Meier survival analysis comparing the OA and the ON group. No statistically significant difference was noted between these two groups (log-rank test *p* = 0.443). On the other hand, no failure was recorded from the ten patients who received lateral UKAs. There were 15 patients with 17 knees who were reported as deceased, one of which sustained implant loosening at post-operative half year received revision surgery; while the others died with intact prosthesis. All of the patients were included in this study, but the end-point was set at the time they were reported as deceased or the time revision surgery was made.

**Table 2:** Parameter analysis for survived group and failed group

Parameters	All (*N* = 337)	Survived (*n* = 305)	Failed (*n* = 32)	P value
**Demographic**				
Age, year, mean ± SD	66.0 ± 8.2	66.3 ± 8.0	63.9 ± 9.7	0.124
Sex, female / male	272 / 65	248 / 57	24 / 8	0.389
Diagnosis, OA / ON	266 / 71	242 / 63	24 / 8	0.566
BMI, kg/m^2^, mean ± SD	27.9 ± 3.7	27.9 ± 3.7	28.3 ± 3.8	0.285
Hypertension	180	162	18	0.735
Diabetes mellitus	48	46	2	0.174
**Femur size**				0.500
A	11	10	1	
B	49	44	5	
C	130	122	8	
D	103	91	12	
E	42	36	6	
F	2	2	0	
**Tibia size**				0.376
1	143	128	15	
2	125	116	9	
3	57	49	8	
4	12	12	0	
**Insert size**	8.8 ± 1.2			0.351
8	202	181	21	
9	41	36	5	
10	74	69	5	
11	6	6	0	
12	12	11	1	
14	2	2	0	
**Radiographic**				
Pre-op aFTA, mean ± SD (°)	-0.7 ± 3.8	-0.6 ± 3.6	-1.7 ± 5.2	0.137
Post-op aFTA, mean ± SD (°)	5.3 ± 3.8	5.4 ± 3.6	4.0 ± 5.0	0.137
ΔaFTA, mean ± SD (°)	6.0 ± 3.0	6.0 ± 3.1	6.1 ± 2.9	0.529
Pre-op PTS, mean ± SD (°)	9.7 ± 4.2	9.7 ± 4.2	9.8 ± 4.1	0.936
Post-op PTS, mean ± SD (°)	7.5 ± 3.8	7.5 ± 3.7	7.9 ± 4.7	0.575
ΔPTS, mean ± SD (°)	4.2 ± 3.3	4.3 ± 3.3	4.2 ± 3.5	0.677
Pre-op aMPTA, mean ± SD (°)	85.6 ± 2.6	85.6 ± 2.5	84.9 ± 2.9	0.149
Post-op aMPTA, mean ± SD (°)	87.8 ± 4.0	87.9 ± 3.5	87.0 ± 4.4	0.212
ΔaMPTA, mean ± SD (°)	3.7 ± 2.6	3.7 ± 2.6	4.4 ± 2.7	0.149

**Table 3:** Survival table of fixed-bearing medial UKA

Year	Number	Withdrawn	Failures	Number at risk	Failure rate (%)	Cumulative survival (%)
0	337	0	6	337.0	1.8	98.2
1	331	0	5	331.0	1.5	96.7
2	326	0	1	326.0	0.3	96.4
3	325	0	1	325.0	0.3	96.1
4	324	0	1	324.0	0.3	95.8
5	323	0	2	323.0	0.6	95.3
6	321	0	7	321.0	2.2	93.2
7	314	0	2	314.0	0.7	92.6
8	312	4	0	310.0	0.0	92.6
9	308	24	1	296.0	0.3	92.2
10	283	13	2	276.5	0.7	91.6
11	268	25	0	255.5	0.0	91.6
12	243	13	3	236.5	1.3	90.4
13	227	14	1	220.0	0.5	90.0
14	212	30	0	197.0	0.0	90.0
15	182	42	0	161.0	0.0	90.0
16	140	53	0	113.5	0.0	90.0
17	87	38	0	68.0	0.0	90.0
18	49	49	0	24.5	0.0	90.0

### Failure reasons

There were 32 failed cases in total. After thorough examination of medical records and radiographs, reason for failure was attributed to five categories, namely implant loosening, osteoarthritis progression, insert wear, infection, and periprosthetic fracture. Details about reasons for revision are listed in Table 4. In this study, all of the loosening cases were related to the tibial component, which could be recognized radiographically with peri-implant radiolucency, and physically with knee pain during activity.

**Table 4:** Reasons of failures

Indication for revision	Procedure	Case numbers	Average year after primary operation
**OA group**		24/266 (9.0%)	6.07
Implant loosening	TKA	9 (37.5%)	5.26
OA progression	TKA	6 (25.0%)	6.25
Insert wear	TKA	5 (20.9%)	8.12
Infection	Two-stage exchange arthroplasty into TKA	2 (8.3%)	9.38
Periprosthetic fracture	TKA with tibial stem	2 (8.3%)	0.75
**ON group**		8/71 (11.3%)	3.21
Implant loosening	TKA	2 (25.0%)	0.96
OA progression	TKA	1 (12.5%)	1.25
Insert wear	TKA	2 (25.0%)	9.80
Infection	Two-stage exchange arthroplasty into TKA	2 (25.0%)	1.38
Periprosthetic fracture	TKA with tibial stem	1 (12.5%)	0.17

### Risk factors analysis

Table 5 showed logistic regression analysis for failure risk factors among different groups. No significant factors were noted in overall survival group and failure group. In the implant loosening group, age and hypertension were significant factors with odds ratio (OR) 0.909 (*p* = 0.017) and 0.179 (*p* = 0.035) respectively. In the insert wear group, post-operative aFTA and ΔPTS also showed significance with OR 0.363 (*p* = 0.019) and 0.415 (*p* = 0.032) individually. While in the periprosthetic fracture group, the post-operative aMPTA was significant factor with OR 0.680 (*p* = 0.045).

**Table 5:** Logistic regression analysis for failure risk factors

Parameters	Univariate P-value	Multivariate P-value	Odds Ratio (95% CI)
**Overall outcome (Survived: 305 / Failed: 32)**			
Bilateral knee operation	0.592	Not included	
Age	0.125	0.275	0.976 (0.933–1.020)
Sex (male as reference)	0.392	Not included	
Diagnosis (ON as reference)	0.567	Not included	
BMI	0.522	Not included	
Hypertension	0.735	Not included	
Diabetes mellitus	0.190	0.143	0.319 (0.069–1.473)
Femur size	0.660	Not included	
Tibia size	0.546	Not included	
Insert size	0.306	Not included	
Pre-op aFTA	0.098	0.967	0.997 (0.885–1.125)
Post-op aFTA	0.050	0.263	0.932 (0.825–1.054)
ΔaFTA (absolute value)	0.834	Not included	
Pre-op PTS	0.924	Not included	
Post-op PTS	0.574	Not included	
ΔPTS (absolute value)	0.906	Not included	
Pre-op aMPTA	0.149	Not included	
Post-op aMPTA	0.212	0.083	0.911 (0.820–1.012)
ΔaMPTA (absolute value)	0.150	0.054	1.163 (0.998–1.356)
**Implant loosening (Survived: 305 / Failed: 11)**			
Age	0.057	0.017	0.909 (0.840–0.983)
Hypertension	0.081	0.035	0.179 (0.036–0.887)
**Insert wear (Survived: 305 / Failed: 7)**			
Post-op aFTA	0.005	0.019	0.363 (0.156–0.848)
ΔPTS (absolute value)	0.024	0.032	0.415 (0.186–0.928)
**Periprosthetic fracture (Survived: 305 / Failed: 3)**			
Post-op aMPTA	0.038	0.045	0.680 (0.467–0.991)

CI, confidence interval

## Discussion

This study demonstrated that patients who underwent medial UKA with a fixed-bearing prosthesis had favorable long-term survivorship. In terms of cumulative survivorship, it was found to be 91.6% at ten years and 90.0% at 15 years. Table 6 summarized the studies of UKA with at least ten-year follow-up [7–10, 13, 14, 16–22]. Differences in implant designs, patient selection and surgical technique made it difficult to compare our results with those of other studies in detail as these may influence individual outcomes. It also appeared that the registry data tend to have inferior outcome. The ten lateral UKAs were excluded from further analysis in this study; however, no failure was recorded in the limited series. Previous study also revealed positive long-term survivorship for the lateral UKAs [23]. The major reason for procedure failure in this study was implant loosening, which is similar to most previous studies. In a systematic review on failures of UKAs, aseptic loosening and OA progression were reported to be the major failure modes [24].

**Table 6:** Review of outcomes of UKA

Study	Prosthesis	Number of knees	Survival at ten years (%)
Murray et al. [9]	Oxford	144	97.7
Vorlat et al. [14]	Oxford	149	82.0
Heck et al. [16]	Marmor & Zimmer	294	91.4
Rajasekhar et al. [19]	Oxford	135	94.0
Naudie et al. [18]	Miller-Galante	113	90.0
Perkins & Gunckle [13]	Zimmer	40	74.0
Kagan et al. [17]	Zimmer	160	87.0
Grave et al. [22]	Zimmer	460	94.2
Scott et al. [21]	Unicondylar	100	85.0
Rossi et al. [20]	Zimmer	148	89.5
Niinimäki et al. [10]	Registry (all)	4713	80.6
Furnes et al. [8]	Registry (all)	2288	80.1
Martino et al. [7]	Registry (all)	6453	87.3

Parameter analysis done on the overall survival group and failure group showed no statistically significant differences in demographic, implant size, and radiographic data (Table 2). In the univariate and stepwise multivariate analysis for overall outcome (Table 5), no single factor was found to have a statistically significant effect. This might be explained by the insufficiency in the failure numbers or the differences in factors pertaining to different failure modes. As a result, subgroup analysis was conducted to determine the potential factors leading to specific failure.

A previous review article concluded that loosening may be determined by under-correction of the deformity, component malalignment, anterior cruciate ligament deficiency, and excessive tibial slope [25]. In this study, however, age and hypertension were two factors that showed statistically significant results in reducing the risk of implant loosening after stepwise multivariate analysis. Concerning the association with age, implant loosening rate was found to be lower in the elderly. This may be related to the serene lifestyles and low activity levels that often exhibited in the elderly patients. Some studies reported that the UKA outcomes were better in the elderly population [13, 19, 22, 26, 27]. According to the data from Australian and Swedish registries, the cumulative revision rate at seven years was 7.5% in patients older than 65 years, compared to 14% in those who were less than 65 years of age [26]. While another study showed that UKAs performed in patients less than 55 years old had an acceptable predicted survivorship of 90.4% at ten years [28]. Concerning the association with hypertension, the current study revealed a relationship between having hypertension and a decreased risk for implant loosening although no large studies confirmed this relationship. In this series, the patients with hypertension all had antihypertensive medication, and it was speculated that patients with hypertension also have exercise load restrictions, which might explain the reduced risk of implant loosening.

Multivariate regression in the insert wear group showed that post-operative aFTA and ΔPTS were potential risk factors. In this study, the post-operative aFTA was 5.4 ± 3.6° in the survival group, which is close to the normal alignment value [29]. Meanwhile, the post-operative aFTA in the insert wear group was 1.4 ± 4.0°, indicating that a post-operative alignment closer to the normal valgus angle reduces the load on the insert. A retrospective study on fixed-bearing medial UKA showed that severe under-correction in varus was associated with an increased insert wear [30]. Additionally, the mean of pre-operative PTS in the survival group was 9.7 ± 4.2° and that of the insert wear group was 9.7 ± 4.0°. The mean ΔPTS and post-operative PTS was 4.3 ± 3.3° and 7.5 ± 3.7° respectively in the survival group, compared to 1.3 ± 1.1° and 9.5 ± 5.0° in the insert wear group. This indicated that if the correction of the PTS was not sufficient, there might be a potential risk of insert wear in the long term, even though the post-operative PTS was not included in the final multivariate analysis model. Weber et al. simulated implant wear on medial fixed-bearing UKA in vitro and concluded that a PTS between 4 and 8°could reduce the wear rate [31]. In the survival group, PTS correction was enough to achieve this range, which is compatible with the results of the simulated study.

Although the group that suffered from a periprosthetic fracture was small, multivariate regression done showed that post-op aMPTA was statistically significant with OR 0.680 (95% CI 0.467–0.991, *p* = 0.045). All of the failed cases in this study suffered from tibial plateau split fractures, where fracture lines were seen to extend from the keel part of the tibial component. Conversion to TKA with tibial stem were made for implant stability and early mobilization of patients. In the survival group, the post-op aMPTA was 87.9 ± 3.5°, as compared to 83.4 ± 1.1° in the fracture group. Based on that, it was supposed that a greater post-op aMPTA may lead to a reduced shearing force on the tibial component during weight-bearing, which subsequently decreases the risk of periprosthetic fractures. Hence, to prevent UKA-related periprosthetic fracture, varus post-operative aMPTA should be avoided. Other previously reported factors that increase the risk of periprosthetic fracture include extended sagittal tibial cuts, low bone mineral density, and large post-op tibiofemoral angle [32, 33]. .

With the advancement in implant designs and surgical techniques, the indications of UKA have been redefined several times [5, 27, 34, 35]. In this study, strict criteria for patient selection such as setting an age limit or a body weight target used in other studies was not applied [27, 34]. Thompson et al. concluded that patients younger than 60 years old and patients with BMI > 35 did have good results with fixed-bearing UKA [35]. However, in the current series, younger age was correlated with an increased risk of implant loosening, while body weight was not. A study by Cavaignac et al. showed that body weight ≧ 82 kg or BMI ≧ 30 were not significantly related to increased failure rate in fixed-bearing UKAs [36]. In contrast, Nettrour et al. found that the rate of early major revision surgery was 5-times more likely in morbidly obese patients whose BMI exceeded 40 [37].

In previously reported studies, UKA for ON had generally favorable results compared with UKA for OA [10–12, 38, 39]. However, in this study, the survivorship of UKA for OA was better than that of UKA for ON, a finding similar to the study by Servien et al. [1]. The difference, however, was not statistically significant (*p* = 0.443). On the other hand, studies revealed that reliable clinical outcomes and survivorship were obtained in UKA for primary osteonecrosis; nevertheless, secondary osteonecrosis was a risk factor for poorer survivorship [38].

This study had some limitations. First, this is a retrospective study. Second, the surgeries were not performed by a single surgeon. The surgery quality, however, is considered to be similar across different surgeons due to the high level of expertise they exhibit at the institution. Third, due to the small sample size of failure numbers, the statistical analysis is lacking in power. To combat that, a discreet process for the multivariate regression model was applied. Since the number was insufficient, the factors that we proposed were potential factors that might serve as a direction for future larger study. Fourth, patient-reported functional outcomes was not available for most cases in the record. However, most patients with regular follow-up showed asymptomatic knee condition. Lastly, there were plenty of patients who were lost to follow-up. The actual survival rate would have improved if these patients had improvements in knee function and no further need for clinic visits were required. The survival rate however could also have been worst if some of these patients went to another hospital for revision surgeries.

## Conclusions

This study showed that fixed-bearing medial UKA provides satisfactory long-term outcome with cumulative survivorship of 91.6% at ten years and 90.0% at 15 years. Tibial component loosening is the major cause of failure. Older age and hypertension were factors associated with decreased risk of implant loosening. With appropriate patient selection and proper surgical technique, a fixed UKA presents a reasonable treatment for patients suffering from isolated compartmental OA or ON of the knee.

### Electronic supplementary material

Below is the link to the electronic supplementary material.

Supplementary Material 1

## Data Availability

The datasets used and/or analyzed during the current study are available from the corresponding author on reasonable request.

## Abbreviations

OA: osteoarthritis
ON: osteonecrosis
UKA: unicompartmental knee arthroplasty
TKA: total knee arthroplasty
BMI: body mass index
aFTA: anatomical femorotibial angle
PTS: posterior tibial slope
aMPTA: anatomical medial proximal tibial angle
OR: odds ratio
