# Supplementary material for: Survivorship of the fixed-bearing medial unicompartmental knee arthroplasty: mean 14-year follow-up in a single medical center
Source: BMC Musculoskelet Disord. 2024 Apr 12;25:283. doi: 10.1186/s12891-024-07378-1 (PMC11010285; doi:10.1186/s12891-024-07378-1)
Supplement: Supplementary file 1 — Supplementary Material 1 [file 12891_2024_7378_MOESM1_ESM.docx]

| Supplementary table of complete logistic regression analysis for risk factors | | | | |
| --- | --- | --- | --- | --- |
| Parameters | | Univariate  P-value | Multivariate  P-value | Odds ratio (95% CI) |
| Overall outcome (Survived: 305 / Failed: 32) |  | |  |  |
| Bilateral knee operation | 0.592 | | Not included |  |
| Age | 0.125 | | 0.275 | 0.976 (0.933-1.020) |
| Sex (male as reference) | 0.392 | | Not included |  |
| Diagnosis (ON as reference) | 0.567 | | Not included |  |
| BMI | 0.522 | | Not included |  |
| Hypertension | 0.735 | | Not included |  |
| Diabetes mellitus | 0.190 | | 0.143 | 0.319 (0.069-1.473) |
| Pre-op aFTA | 0.098 | | 0.967 | 0.997 (0.885-1.125) |
| Post-op aFTA | 0.050 | | 0.263 | 0.932 (0.825-1.054) |
| ΔaFTA (absolute value) | 0.834 | | Not included |  |
| Pre-op PTS | 0.924 | | Not included |  |
| Post-op PTS | 0.574 | | Not included |  |
| ΔPTS (absolute value) | 0.906 | | Not included |  |
| Pre-op aMPTA | 0.149 | | Not included |  |
| Post-op aMPTA | 0.212 | | 0.083 | 0.911 (0.820-1.012) |
| ΔaMPTA (absolute value) | 0.150 | | 0.054 | 1.163 (0.998-1.356) |
| Implant loosening group (Survived: 305 / Failed: 11) | | | | |
| Bilateral knee operation | | 0.998 | Not included |  |
| Age | | 0.057 | 0.017 | 0.909 (0.840-0.983) |
| Sex (male as reference) | | 0.966 | Not included |  |
| Diagnosis (ON as reference) | | 0.842 | Not included |  |
| BMI | | 0.495 | Not included |  |
| Hypertension | | 0.081 | 0.035 | 0.179 (0.036-0.887) |
| Diabetes mellitus | | 0.588 | Not included |  |
| Pre-op aFTA | | 0.925 | Not included |  |
| Post-op aFTA | | 0.739 | Not included |  |
| ΔaFTA (absolute value) | | 0.464 | Not included |  |
| Pre-op PTS | | 0.458 | Not included |  |
| Post-op PTS | | 0.798 | Not included |  |
| ΔPTS (absolute value) | | 0.150 | 0.104 | 1.158 (0.970-1.381) |
| Pre-op aMPTA | | 0.474 | Not included |  |
| Post-op aMPTA | | 0.983 | Not included |  |
| ΔaMTPA (absolute value) | | 0.429 | Not included |  |
| OA progression group (Survived: 305 / Failed: 7) | | | | |
| Bilateral knee operation | | 0.815 | Not included |  |
| Age | | 0.700 | Not included |  |
| Sex (male as reference) | | 0.514 | Not included |  |
| Diagnosis (ON as reference) | | 0.682 | Not included |  |
| BMI | | 0.696 | Not included |  |
| Hypertension | | 0.833 | Not included |  |
| Diabetes mellitus | | 0.998 | Not included |  |
| Pre-op aFTA | | 0.267 | Not included |  |
| Post-op aFTA | | 0.137 | 0.553 | 0.942 (0.774-1.147) |
| ΔaFTA (absolute value) | | 0.821 | Not included |  |
| Pre-op PTS | | 0.566 | Not included |  |
| Post-op PTS | | 0.867 | Not included |  |
| ΔPTS (absolute value) | | 0.409 | Not included |  |
| Pre-op aMPTA | | 0.036 | 0.139 | 0.762 (0.532-1.092) |
| Post-op aMPTA | | 0.861 | Not included |  |
| ΔaMTPA (absolute value) | | 0.191 | 0.490 | 1.098 (0.842-1.433) |
| Insert wear group (Survived: 305 / Failed: 7) | | | | |
| Bilateral knee operation | | 0.389 | Not included |  |
| Age | | 0.054 | 0.686 | 0.976 (0.869-1.097) |
| Sex (male as reference) | | 0.514 | Not included |  |
| Diagnosis (ON as reference) | | 0.613 | Not included |  |
| BMI | | 0.217 | 0.779 | 1.041 (0.788-1.373) |
| Hypertension | | 0.593 | Not included |  |
| Diabetes mellitus | | 0.998 | Not included |  |
| Pre-op aFTA | | 0.006 | 0.078 | 2.092 (0.921-4.752) |
| Post-op aFTA | | 0.005 | 0.019 | 0.363 (0.156-0.848) |
| ΔaFTA (absolute value) | | 0.638 | 0.087 | 2.311 (0.884-6.041) |
| Pre-op PTS | | 0.999 | Not included |  |
| Post-op PTS | | 0.168 | Not included |  |
| ΔPTS (absolute value) | | 0.024 | 0.032 | 0.415 (0.186-0.928) |
| Pre-op aMPTA | | 0.043 | Not included |  |
| Post-op aMPTA | | 0.013 | 0.118 | 0.826 (0.651-1.049) |
| ΔaMTPA (absolute value) | | 0.534 | Not included |  |
| Periprosthetic joint infection group (Survived: 305 / Failed: 4) | | | | |
| Bilateral knee operation | | 0.998 | Not included |  |
| Age | | 0.900 | Not included |  |
| Sex (male as reference) | | 0.749 | Not included |  |
| Diagnosis (ON as reference) | | 0.183 | 0.331 | 0.363 (0.047-2.801) |
| BMI | | 0.137 | 0.175 | 0.824 (0.623-1.090) |
| Hypertension | | 0.292 | Not included |  |
| Diabetes mellitus | | 0.589 | Not included |  |
| Pre-op aFTA | | 0.864 | Not included |  |
| Post-op aFTA | | 0.753 | Not included |  |
| ΔaFTA (absolute value) | | 0.895 | Not included |  |
| Pre-op PTS | | 0.873 | Not included |  |
| Post-op PTS | | 0.906 | Not included |  |
| ΔPTS (absolute value) | | 0.393 | Not included |  |
| Pre-op aMPTA | | 0.798 | Not included |  |
| Post-op aMPTA | | 0.187 | 0.195 | 1.257 (0.890-1.776) |
| ΔaMTPA (absolute value) | | 0.369 | Not included |  |
| Periprosthetic fracture group (Survived: 305 / Failed: 3) | | | | |
| Bilateral knee operation | | 0.998 | Not included |  |
| Age | | 0.304 | Not included |  |
| Sex (male as reference) | | 0.529 | Not included |  |
| Diagnosis (ON as reference) | | 0.579 | Not included |  |
| BMI | | 0.945 | Not included |  |
| Hypertension | | 0.506 | Not included |  |
| Diabetes mellitus | | 0.998 | Not included |  |
| Pre-op aFTA | | 0.728 | Not included |  |
| Post-op aFTA | | 0.199 | 0.787 | 0.943 (0.617-1.442) |
| ΔaFTA (absolute value) | | 0.064 | 0.092 | 0.566 (0.292-1.097) |
| Pre-op PTS | | 0.476 | Not included |  |
| Post-op PTS | | 0.769 | Not included |  |
| ΔPTS (absolute value) | | 0.865 | Not included |  |
| Pre-op aMPTA | | 0.745 | Not included |  |
| Post-op aMPTA | | 0.038 | 0.045 | 0.680 (0.467-0.991) |
| ΔaMTPA (absolute value) | | 0.532 | Not included |  |

CI, confidence interval; OA, osteoarthritis; ON, osteonecrosis; BMI, body mass index; aFTA, anatomical femorotibial angle; PTS, posterior tibial slope angle; aMPTA, anatomical medial proximal tibial angle; Δvalues were calculated by subtracting the preoperative values from the postoperative values (in absolute values)
